# Prevalence and correlates of self-reported state of teeth among schoolchildren in Kerala, India

**DOI:** 10.1186/1472-6831-6-10

**Published:** 2006-07-03

**Authors:** Jamil David, Anne N Åstrøm, Nina J Wang

**Affiliations:** 1 Department of Oral Sciences – Pedodontics, Årstadveien 17, NO-5009 Bergen, Norway; 2 Centre for International Health, Armauer Hansen Building, NO-5021 Bergen, Norway; 3 Institute of Clinical Dentistry – Pedodontics, P.O. Box 1109 Blindern, NO-0317 Oslo, Norway

## Abstract

**Background:**

Oral health status in India is traditionally evaluated using clinical indices. There is growing interest to know how subjective measures relate to outcomes of oral health. The aims of the study were to assess the prevalence and correlates of self-reported state of teeth in 12-year-old schoolchildren in Kerala, India.

**Methods:**

Cross-sectional survey data were used. The sample consisted of 838 12-year-old schoolchildren. Data was collected using clinical examination and questionnaire. The clinical oral health status was recorded using Decayed, Missing and Filled Teeth (DMFT) and Oral Hygiene Index – Simplified (OHI-S). The questionnaire included questions on sociodemographics, self reports of behaviour, knowledge and oral problems and a single-item measuring self-reported state and satisfaction with appearance of teeth. The Kappa values for test-retest of the questionnaire ranged from 0.55 to 0.97.

**Results:**

Twenty-three per cent of the schoolchildren reported the state of teeth as bad. Multivariate logistic regression showed significant associations between schoolchildren who reported to have bad teeth and poor school performance (Odds Ratio (OR) = 2.5), having bad breath (OR = 2.4), food impaction (OR = 1.7) dental visits (OR = 1.6), being dissatisfied with appearance of teeth (OR = 4.2) and caries experience (OR = 1.7). The explained variance was highest when the variables dental visits, bleeding gums, bad breath, food impaction and satisfaction with appearance were introduced into the model (19%).

**Conclusion:**

A quarter of 12-year-olds reported having bad teeth. The self-reported bad state of teeth was associated with poor school performance, having bad breath and food impaction, having visited a dentist, being dissatisfied with teeth appearance and having caries experience. Information from self-reports of children might help in planning effective strategies to promote oral health.

## Background

Oral health is fundamental to general health and well being [1]. From a theoretical point of view, three major dimensions of oral health has been identified; clinically assessed disease and impairment, disease and treatment specific symptoms and functional and psychological disabilities [2]. It is now widely accepted that in addition to clinical indicators, functional, social and psychological aspects of oral health status should be considered when assessing dental needs [3,4]. Several subjective oral health indicators have been developed to assess functional, social and psychological oral health outcomes ranging from single item global indicators, such as satisfaction with oral health and satisfaction with appearance of teeth, to complex inventories and scoring systems [5]. In dentistry, many multi-item scales have been applied, but single item indicators have shown to be advantageous and is widely used in oral health research [6]. Cunny and Perri [7] suggest that when operational costs tend to increase, single-item indicators might be appropriate for use as they are strongly correlated with multi-item scales.

The majority of subjective oral health indicators have been used to evaluate oral health outcomes in adult populations [8,9]. Oral health outcomes in children have also been explored [10-13]. According to recent reports, age-specific questionnaires are valid and reliable instruments for assessing oral health outcomes in children [14-16]. In this study information on subjective oral health was achieved by introducing a questionnaire to 12-year-old schoolchildren. By this age, children are thought to have matured enough to report on oral health and influencing factors [17].

Reisine and Bailit [18] suggested that age, gender, social class and clinical status may be important variables in understanding how an individual perceives his/her oral health status. It is evident, for instance, that girls perceive their oral health more positively than boys [19], but tend to be less satisfied with the appearance of teeth [19]. Subjects of higher socio-economic status (SES) tend to be more satisfied with oral health than lower SES counterparts [20,21], whereas dental pain has been reported to be most prevalent in families of lower income and education [22,23]. On the other hand, schoolchildren resident in urban areas have been found to be more dissatisfied with oral health than those from rural areas [24]. Gherunpong et al. [25] and Marshman et al. [26] provided evidence that bleeding gums and number of missing teeth impacted the oral health related quality of life of schoolchildren. Oral problems such as bad breath and bleeding gums have been identified to impact on students' perceived health and well-being [25,27].

Few attempts have been made to assess the prevalence and socio-behavioural determinants of children's perceived oral health status in developing countries such as India. This is notable, since children experience more oral impacts than adults [25]. Children who have poor oral health have been found to be 12 times more likely to have restricted activity days than those who do not [28]. As developing countries have limited resources allocated for oral health services, as for instance in India where less than seven percent of the gross national product is spent on health care, it is anticipated that self-reports can be utilized together with clinical indicators to assess the need for dental care [29]. In this study, self-reported state of teeth refers to the child's present opinion regarding his or her state of teeth as good or bad. The aims of the present study were to assess the prevalence and correlates of self-reported state of teeth in 12-year-old schoolchildren in Kerala, India.

## Methods

### Sample and data collection

The study population consisted of 12-year-old schoolchildren attending private and government upper primary schools (Grade 7) in urban and rural areas of Thiruvananthapuram district. A stratified, two stage random cluster sample design was applied, using schools as the primary sampling unit. The sample size was estimated allowing for a design factor of 2, caries prevalence of 60% [29] and precision of 0.05. The required sample size calculated was 738. Fifteen percent was added in order to counter non-response. At stage 1, 30 schools (8 urban from a total of 39 and 22 rural from a total of 177) were selected with probability proportional to size from the list of schools in the areas. At stage 2, 28 schoolchildren were randomly selected from each school selected at stage 1 on the day of the examination. Twenty-eight children were not available in three schools. In schools where 28 children were not found, efforts were made to get schoolchildren from other schools in the same area. This yielded a sample size of 838. Data were collected by questionnaire and clinical examination.

### Questionnaire

The questionnaire was constructed and administered in English. After a pilot study, the questionnaire was translated into the local language (Malayalam) using appropriate and simple words. For validation the questionnaire was translated back into English. During the survey the questions were read to the schoolchildren one by one providing them with ample time to answer the questions. Teachers were not present in the classrooms when children answered the questionnaire.

### Dependent variable

*Self-reported state of teeth *was assessed using a single question. "What do you think is the state of your teeth?" A four-point scale (1) very good, (2) good, (3) bad, (4) very bad was initially used in the questionnaire and collapsed into a dichotomous variable, (0) good teeth (including original categories 1, 2) and (1) bad teeth (including original categories 3, 4) in the analyses.

### Independent variables

Family wealth was assessed as an indicator of socio-economic status using a modified version of the standard approach used in equity analysis [30]. Household durable assets indicative of family wealth (e.g. bicycle, television, fridge, motorcycle, car) were assessed as (0) No and (1) Yes. A sum family wealth index was constructed (range 0–17) and categorised as (0) 0 = poor class, (1) 1–10 = middle class and (2) 11–17 = high class. *School performance *was assessed by one question: "In your opinion, what does your class teacher think about your school performance compared to that of your classmates?" The variable was categorized as (0) good school performance and (1) poor school performance. *Self-reported oral problems *were assessed by four questions, "Have you ever had bleeding gums, bad breath, toothache or food impaction?" The answers were categorised as (0) no and (1) yes. *Dental visits *were assessed by the question: "Have you ever visited a dentist?" The answers were categorized as (0) no and (1) yes. *Oral health knowledge *was assessed based on answers to statements related to tooth brushing, sugar, preventive role of fluoride, attendance to the dentist, tobacco's association with oral cancer and gum disease and role of genetics in acquiring unhealthy teeth. The answers were summed and categorised as follows: (0) 0–4 score = poor knowledge and (1) 5–9 score = good knowledge. *Satisfaction with appearance of teeth *was assessed by the question: "How satisfied or dissatisfied are you with the appearance of your teeth?" A four-point scale (1) very satisfied, (2) satisfied, (3) dissatisfied, (4) very dissatisfied was initially used in the questionnaire and categorized as, (0) satisfied with appearance with teeth (including original categories 1, 2) and (1) dissatisfied with appearance with teeth (including original categories 3, 4).

### Clinical examination

The clinical examination was carried out in the classroom for all the children by one dentist (JD) who was assisted by a trained recorder. The dentist carried out calibration exercises at the Department of Pediatric Dentistry, Faculty of Dentistry, Bergen, before the study was performed. Oral Hygiene Index – Simplified (OHI-S) was used to evaluate the oral hygiene status [31]. Number of fractured anterior teeth was recorded. The criteria described by WHO was used to record dental caries [32]. During the survey, torchlight was used for illuminating the oral cavity. No radiographs were taken and drying of the teeth was not performed. In each school two children were randomly re-introduced for oral examination by the recorder to analyze intra-examiner reliability. Details of the clinical examination are reported in a previous publication [33].

### Statistical analyses

The sample size was calculated using the statistical software packages EPI INFO ™ version 6 and the data analysed using SPSS version 13.0 (SPSS Inc, Chicago IL). Bivariate results were tested using chi-square statistics. A stepwise multiple logistic regression analysis was performed with self-reported state of teeth as the dependent variable. Two-way interactions were checked in multiple logistic regression analysis. To control for potential confounding, gender, area, socioeconomic status and school performance were forced into step 1 of the multivariate analysis independent of bivariate statistical significance. In step 2 questions related to self-reported oral problems (bleeding gums, bad breath and food impaction) behavioural (dental visits) and satisfaction with appearance of teeth were entered. In step 3 caries experience and oral hygiene status were included. The 95% confidence intervals (CI) and odds ratios (ORs) were estimated to determine the significance of the predictor variables. Intra-examiner reliability of dental caries examination and the reliability of the test-retest of the questionnaire are reported using Cohen's kappa. The significance level was set at 5%. To adjust for potential cluster effects, analyses were conducted with STATA (9.0). This analysis showed that the initial results of unadjusted analyses were left essentially unchanged when cluster effects were taken into account.

### Ethical permission

Permission was given from the Ethical Committee at the Thiruvananthapuram Medical College, the Norwegian Ethical Committee and the Directorate of Public Instruction, Kerala. Written consent was given from the head of the school and the participating children.

## Results

### Test – retest reliability

A total of 108 schoolchildren were selected from the participants of the survey to test the reliability of the questionnaire. The interval between the test and retest ranged from 7 to 19 days. Kappa values for test-retest of the questionnaire ranged from 0.55 (knowledge) to 0.97 (wealth index). These values are in the interval from moderate to substantial agreement according to Landis and Koch [34]. The intra-examiner reliability value for caries examination was considered to almost perfect with a kappa value of 0.88 [34].

A total of 838 school children, 57% boys participated in the study. The majority of the participants were rural residents and had a middle socio-economic background. A total of 27% of the schoolchildren investigated had caries experience (DMFT > 0) and 23% reported the state of teeth to be bad (Table 1).

**Table 1:** Distribution of 12-year-old schoolchildren according to dependent and independent variables.

Dependent variable	Categories	Number (%)
State of teeth	Good	644 (77)
	Bad	194 (23)

Independent variables		

Gender	Girls	359 (43)
	Boys	479 (57)
Place of residence	Rural	616 (74)
	Urban	222 (26)
Socio-economic status	Poor	212 (25)*
	Middle class	585 (70)
	High class	40 (5)
School performance	Good	681 (81)
	Poor	157 (19)

Bleeding gums	No	143(17)
	Yes	695 (83)
Bad breath	No	547 (65)
	Yes	291 (35)
Toothache	No	266 (32)*
	Yes	571 (68)
Food impaction	No	239 (29)
	Yes	599 (71)
Dental visits	Never	504 (60)
	Yes	334 (40)
Satisfied with appearance of teeth	Satisfied	526 (63)
	Dissatisfied	312 (37)
Oral health knowledge	Good	487 (59)*
	Poor	344 (41)

Caries experience	DMFT = 0	612 (73)
	DMFT > 0	226 (27)
Oral hygiene index	Good	681 (81)
	Fair	157 (19)
Anterior teeth fracture	No	787 (94)
	Yes	51 (6)

* The totals of the numbers in the categories do not add up to 838 because of missing data

Table 2 shows the number and percentage of 12-year-olds with caries experience and self-reported bad state of teeth by gender and area of residence. The proportion of children with caries experience (DMFT > 0) was higher in urban than in rural areas (33% versus 25%, p < 0.05). Children living in urban areas also tended to report having bad state of teeth as compared to those living in rural areas (28% versus 21%, p < 0.05). A higher proportion of girls than boys in rural areas reported to have bad state of teeth (25% versus 17%) (p < 0.05).

**Table 2:** Number (%) of 12-year-old schoolchildren with caries experience and self-reported bad state of teeth according to area of residence and gender.

	DMFT > 0 n (%)	Bad state of teeth n (%)
Urban		
all	74 (33)*	63 (28)*
girls	34 (38)	35 (27)
boys	40 (30)	28 (31)

Rural		
all	152 (25)	131 (21)
girls	66 (25)	86 (25)†
boys	86 (25)	45 (17)

* Chi-square test, p < 0.05 (comparison between urban and rural)

† Chi-square test, p < 0.05 (comparison between girls and boys in rural area)

Table 3, depicts the proportions of 12-year-old schoolchildren reporting bad teeth status according to various independent variables and the adjusted odd ratios and 95% confidence intervals from multiple logistic regression analyses. Multivariate analysis showed that children who reported bad school performance, bad breath, food impaction, and caries experience and those visiting a dentist and being dissatisfied with appearance of teeth were more likely to report bad teeth status than their counterparts in the opposite groups (Table 3). The children most likely to report bad state of teeth were children dissatisfied with appearance of the teeth (OR = 4.2), children who reported poor school performance (OR = 2.5) and children who reported bad breath (OR = 2.4). The independent variables were checked for interactions, but none were identified. Socio-demographic and school performance variables explained 7% (Nagelkerke's R^2 ^= 0.07, Model Chi-Square 7.9, df = 6, p > 0.05) of the variance in children's self-reported state of teeth. Including behavioural, self-reported oral problems and reported satisfaction with appearance of teeth increased the explainable variance to 26% (Nagelkerke's R^2 ^= 0.26, Model Chi-Square 20.1, df = 8, p > 0.05). Taking into account clinical indicators (dental caries and oral hygiene status) the total variance explained by all the factors analysed was 27% (Nagelkerke's R^2 ^= 0.27, Model Chi-Square 4.5, df = 8, p > 0.05).

**Table 3:** Number (%) of schoolchildren who reported the state of teeth to be bad by socio-behavioural factors, non-clinical and clinical oral health indicators. Cross-tabulation analysis (chi-square) and multiple logistic regression with odds ratios (OR) and 95% confidence interval (CI).

	Unadjusted	Adjusted		
Independent variables	Bad state of teeth n (%)	OR	95% CI	R^2^

**Step 1**				

Girls	73 (20)	1		
Boys	121 (25)	1.1	0.8–1.6	

Rural	131 (21)*	1		
Urban	63 (28)	1.3	0.9–2.0	

Socio-economic status – Poor	52 (25)	1		
Socio-economic status – Middle class	133 (23)	0.8	0.5–1.2	
Socio-economic status – High class	9 (22.5)	0.3	0.4–2.4	

School performance – Good	130 (19)*	1		
School performance – Poor	64 (41)	**2.5**	**1.6–3.8**	**0.07**

**Step 2**				

Bleeding gums – No	135 (21)*	1		
Bleeding gums – Yes	59 (29)	1.1	0.7–1.7	

Bad breath – No	87 (16)*	1		
Bad breath – Yes	107 (37)	**2.4**	**1.7–3.5**	

Toothache – No	55 (21)			
Toothache – Yes	139 (24)			

Food impaction – No	34 (14)*	1		
Food impaction – Yes	160 (27)	**1.7**	**1.1–2.7**	

Dental visits – Never	97 (19)*	1		
Dental visits – Yes	97 (29)	**1.6**	**1.1–2.3**	

Oral health knowledge – Good	102 (21)			
Oral health knowledge – Poor	90 (26)			

Satisfied with appearance of teeth	68 (13)*	1		
Dissatisfied with appearance of teeth	126 (40)	**4.2**	**2.9–6.0**	**0.26**

**Step 3**				

DMFT = 0	120 (20)*	1		
DMFT > 0	74 (32)	**1.7**	**1.1–2.5**	

Oral hygiene – Good	147 (22)*	1		
Oral hygiene – Fair	47 (30)	1.4	0.9–2.3	

Anterior trauma – no	182 (23)			
Anterior trauma – yes	12 (24)			**0.27**

* p < 0.05

All variables in Step 1 and other statistically significant bivariate variables were entered into the multiple logistic regression analysis

## Discussion

Nearly one-fourth (23%) of the 12-year-old schoolchildren reported having bad teeth. Self-reported state of teeth was significantly associated with poor school performance, self-reported oral problems in terms of bad breath and food impaction, dental visits, dissatisfaction with appearance of teeth and having caries experience (Table 3). Similar findings have been reported elsewhere in terms of social and behavioural factors impacting on adult's as well as on schoolchildren's self-reported oral health [35]. The prevalence of impaired oral health assessed here falls below what has been obtained with multi-item indicators in previous studies from developing and developed countries [20,25,36]. The low prevalence of self-reported bad state of teeth accords with the caries prevalence observed in this study population. Compared to the European average DMFT score of 2.6 in 12-year-olds, the present DMFT score of 0.45 is low [33]. It compares, however with findings from other developing countries in that a high proportion (91%) of the DMFT score was attributable to untreated caries [33]. A majority of the children (81%) investigated showed good oral hygiene, although 83% of the pupils confirmed experience with bleeding gums (Table 1).

The structured questionnaires applied in this study might have certain limitations [37]. Reporting bias due to giving socially desirable answers and lack of recall are frequently encountered by children [38]. Thus, the percentage of children reporting bad state of teeth may have been underestimated [38], because of socially desirable answers or the fact that children were reluctant to express negative opinions and attitudes. Alternatively, a global single item measure of oral health as used in this study might not have been sensitive enough to determine differences in state of teeth scores. Nevertheless, the positive associations between self-reported state of teeth, clinical dental status and self-reported oral problems accords with results from other studies [10,39] and with theory, thus supporting the validity of the single item self-reported oral health indicator used in this study. According to theoretical models [4], impairments refer to the immediate biophysical outcomes of disease, commonly assessed by clinical indicators. Functional limitations, pain and discomfort constitute the earliest negative impacts, which in turn are followed by oral disadvantage and individual's overall assessment of oral health status. Reproducibility scores of the dental caries examination and of the questionnaire items were acceptable. The reliability was strengthened by translating the questionnaire into the local language and consequently ensuring cross-cultural adaptation and validation.

Evidence suggests that children and adults belonging to wealthy families, in terms of education and economic status, tend to have less impaired oral health than their poorer counterparts [20,40,41]. Nicolau et al., [42], have suggested that lower socio-economic status and family living conditions affect school performance and oral health behaviour. School performance was included in multiple logistic regression analysis along with the sociodemographic variables as it has been acknowledged that school progress shows a positive gradient with material possessions [42]. In this study, children who performed poorly in school were more likely to report their teeth status to be bad when compared to subjects who considered that they performed well in school. Although the question regarding school performance was judged according to schoolchildren's own view rather than to their actual grades, it seems surprising to find one-fifth claiming to have performed poorly. It was anticipated that on being questioned about themselves the children would provide positive remarks [38], which does not seem to be the case in this study. The reported bad school performance might be a reflection of children's general state of life [43] as well as of their bad state of teeth.

Consistent with findings in previous studies [18,25,44], the present results revealed positive associations between self-reported state of teeth and dental caries and self-reported oral problems. Studies should be done to see whether perceived oral health status could be improved through strengthening of preventive and therapeutic dental services for primary school children. Gherunpong et al., [25] found that gingival inflammation and bleeding impacted negatively on children's oral quality of life and subsequently prevented them from brushing their teeth. Whereas numerous studies have identified a gap between professionally – and self-defined oral health [45] others have found statistically significant associations of various strength [46]. Thus, the present finding also supports previous studies suggesting that caries experience is a consistent clinical correlate of adolescent's oral quality of life [23,46,47]. The positive association between DMFT scores and self-reported state of teeth might be attributed partly to a high level of untreated dental caries and a high level of unmet need for dental care and partly to a high level of awareness and self-perception of dental disease on the part of the children investigated. Contrary to the results reported by Ostberg et al., [13], the DMFT index was possibly sensitive enough to be associated with self-reported state of teeth even in the presence of low mean DMFT scores.

It is noteworthy that schoolchildren who had experience with dental visits, reported to have bad state of teeth more often than their counterparts with no dental visits. Similar results have been reported previously in developing countries and might reflect symptomatic dental visiting habits and need for emergency care rather than an unexpected response to dental treatment [24,48].

Children who were dissatisfied with their appearance of teeth tended to perceive their teeth status as bad. Earlier studies have reasoned children to be dissatisfied with dental appearance in the presence of fractured anterior teeth, malpositioned teeth and untreated malocclusion [21,25,49]. Although children in the present study had fractured anterior teeth, no significant difference was found between those with and without anterior trauma when reporting the state of their teeth as bad. This type of difference in self-perception might be predisposed by socio-cultural variations. Further investigation might be required to assess the impact of malpositened teeth and malocclusion on self-reported state of teeth.

## Conclusion

This study revealed that 23% of schoolchildren reported the state of teeth to be bad and more so among children with poor school performance, having bad breath and food impaction, those who visited a dentist, were dissatisfied with appearance of teeth and had caries experience. Apart from professionally assessed dental status, self-reports measuring oral health may also play a significant part in gathering information about dental health of children. Information on self-reported oral health may help oral health planners to plan preventive programmes with limited resources [3,39].

## Competing interests

The author(s) declare that they have no competing interests.

## Authors' contributions

JD carried out the data collection, data analysis and writing of the article. ANA initiated the idea and along with NJW supervised the project and assisted in writing/editing of the article. All authors have read and approved the final manuscript.

## Pre-publication history

The pre-publication history for this paper can be accessed here:


